# Learning from past and current food security efforts and challenges in Zimbabwe: The years 1430–2020

**DOI:** 10.4102/jamba.v14i1.1210

**Published:** 2022-09-27

**Authors:** Sifelani Ngwenya, Wilfred Lunga, Elize S. van Eeden

**Affiliations:** 1 Africa Centre Disaster Studies, Faculty of Natural Sciences, North-West University, Potchefstroom, South Africa; 2 Human Sciences Research Council, Pretoria, South Africa; 3 Centre for Disaster Studies, North-West University, Potchefstroom, South Africa; 4 School of Social Sciences, Faculty of Social Sciences, North-West University, Vanderbijlpark, South Africa

**Keywords:** food security, food insecurity, precolonial, colonial, postcolonial, challenges

## Abstract

Zimbabwe has been experiencing food insecurity for many centuries. This study sought to explore and learn from Zimbabwe’s past and current food security (FS) efforts and challenges, through three historical periods, namely the precolonial, colonial and postcolonial, from about 1430 to 2020. The year 1430 marks the establishment of the Monomotapa state, one of the starting points for Zimbabwe’s own national reconstruction. Adopting a qualitative paradigm, data were obtained using document review and interviewing 85 purposively selected key informants, some of whom were found using snowballing. The study found that the adopted FS strategies during the precolonial, colonial and postcolonial periods were dynamic and mainly derived by new political agendas and crises. The food production and storage aspects of the colonial period were built around agricultural extension services and Grain Marketing Board strategies. The postcolonial period FS initiatives pivoted on humanitarian and development programs. Zimbabwe’s FS initiatives across the three historical periods remain susceptible to various challenges (droughts, political antagonism, bureaucracy, partisanship, corruption, incapacitation and weak support systems). As such, Zimbabwe’s food insecurity levels remain far away from being a reality, unless the identified challenges are taken head-on by all stakeholders. Therefore, the study recommends that informed local wisdom be given space in finding a lasting solution to food insecurity. Meanwhile, multistakeholder inclusivity, knowledge development and management should be made the crux of FS-related initiatives. This could foster new partnerships and encourage the ethic of working together and participation towards ensuring FS.

## Introduction

Worldwide, efforts to ensure food security (FS) are an old phenomenon, intertwined with humanity’s struggles with disaster risks that negatively impact livelihoods. Food security efforts date back to folklore, legends and religious stories (Van Niekerk 2005). Oliver and Atmore (1975) assert that the legendary Mutota escaped the Great Zimbabwe kingdom in search of food, reaching a land where he established his kingdom in Northern Zimbabwe and parts of Southern Zambia. The Bible (1995) indicates that Joseph predicted 7 years of plenty and 7 years of famine in Genesis 37:25–36. Massive investment in FS denotes the value and benefits humans attach to food. Food insecurity compromises communities’ well-being. The purpose of the study is to interrogate the precolonial, colonial and postcolonial periods’ FS initiatives from 1430 to 2020. Various international and national strategies employed to ensure and secure FS for all people are brought into the limelight.

The Southern African Development Community (SADC) region has always committed to ensuring FS for its 280 million inhabitants (Muimba-Kankolongo 2018). In pursuit of the same agenda, the Government of Zimbabwe (GOZ) and its partners have designed and implemented various FS initiatives and policies. However, the effort to reduce food insecurity has not yielded much success (Lunga & Musarurwa 2016). There is a continued increase in the number of hungry and food-insecure people in developing countries (Food and Agriculture Organization of the United Nations [FAO] 2019). The lack of progress undermines the immense challenge of achieving the Zero Hunger target by 2030. Several questions about the lack of impact of the GOZ and non-governmental organisations’ (NGOs) efforts to address FS require answers. Past to current FS practices and challenges are interrogated to draw lessons, best practices and recommendations to inform new programs and assessment frameworks.

### Conceptualising food security (insecurity)

Food security and insecurity have immediate impact on how vulnerable communities manage their resources and their social lives. Stakeholders have developed and implemented various disaster-risk reduction (DRR) strategies and initiatives to mitigate food insecurity-induced calamities. Hence, the understanding of concepts is critical. Food security has been subjected to an array of transdisciplinary debates and given approximately 200 definitions over the years (Ignowski 2012). Maxwell and Frankenberger (1992) define FS as a secure access to sufficient food for a healthy life. FS denotes a situation when an entity has physical, social and economic access to sufficient, safe and nutritious food to meet their dietary needs and food preferences (FAO et al. 2017). It is a situation of all-time sufficiency of nutritious food, anchored on pillars of availability, access, utilisation and stability. Therefore, the absence of any one of the pillars renders an entity food insecure. Food and Agriculture Organization (FAO) et al. (2018) go further to state that food insecurity denotes insufficient physical and economic access to safe, nutritious and culturally acceptable food for a healthy and active life. Several DRR strategies have been adopted in various countries to deal with FS and insecurity. Zimbabwe has done its fair share in the three historical periods to ensure FS and manage food insecurity. The following sections provide brief discussions on FS initiatives in Zimbabwe’s three historic periods: the precolonial, colonial and postcolonial.

### Zimbabwe’s past and current food security efforts

Mlambo (2014) provides insights into Zimbabwe’s food initiatives in accordance to the precolonial, colonial and postcolonial historical periods. During these historical periods, different peoples, kingdoms and institutions inhabited the land (Mlambo 2014). These periods had varying influences on communities, giving rise to the formation of new sociopolitical and economic formations, identities, commodities, languages, ideologies, relationships, political and economic outlooks and tastes (GOZ 2020). Lessons can be drawn from processes, events and actions that took place and affected communities in various dimensions.

### Precolonial period (1430–1885)

The precolonial period, according to Mazarire (2008), is a starting point for Zimbabwe’s own national reconstruction. It is known as the pre-1885 period before the scramble for Africa (Heldring & Robinson 2013). Mazarire (2008) and GOZ (2020) posit that this period saw the rise and fall of Great Zimbabwe, the Mutapa, the Torwa, the Rozvi and Ndebele states’ empires. The precolonial Zimbabwe was a multi-ethnic society punctuated by complex, dynamic, fluid and always-changing political, social and economic relations. The most outstanding empires that need mention are the Monomotapa and the Ndebele, characterised by various forms of interaction and with not enough influx of capital to satisfy the needs of the people (Beach 1999). The Monomotapa dynasty stretched over vast areas of approximately 700 000 km^2^, between 1430 AD and 1760 AD (Oliver & Atmore, 1975; eds. Raftopoulos & Mlambo 2008). The Ndebele dynasty was formed by a Nguni fragment of the Zulu kingdom in 1840, whose influence spread from the Limpopo and Zambezi rivers to the north and south and between the Makgadikgadi salt pans to the west and the realm of Soshangana to the east, the Save river (Ndlovu-Gatsheni 2009).

The Monomotapa dynasty used crop diversification, shifting cultivation, food storage and preservation, raiding, ranching, tribute collection, trading and hunting as its main food strategies to ensure the kingdom’s FS (Maruve & Chitongo 2017). Crops grown included maize, beans, pumpkins, sweet reeds and tubers, amongst others. Chirimuuta and Mapolisa (2011) explain that crop diversification and rotation strategies helped improve soil fertility, a good medium for good products that addressed the availability pillar of the FS. The storage and preservation strategies based on indigenous knowledge augmented other strategies (Chirimuuta & Mapolisa 2011) to safeguard food and extend the shelf life of food. Storage facilities were strategically designed to achieve the goal of preserving seasonal and perishable foods like meat, vegetables, fresh maize and seasonal food products. Some food commodities were boiled, dried, stored and preserved using smooth ash and tree leaves (Chirimuuta & Mapolisa 2011; Matutu 2017). Cattle rearing ensured FS, as it played a fundamental role in sustaining lives (Ndlovu-Gatsheni 2009). Cattle provided draught power, meat, milk, manure and transport and were used in marriage issues (*lobola*) and as food during ritual ceremonies (Magama 2014). Other economic activities that helped were trade (exchange of commodities), mining of gold to trade with the Persians, whilst iron was forged into iron tools which enhanced agricultural productivity. Gathering, practiced by women, and hunting by men were other key economic activities in Great Zimbabwe (Magama 2014). Elephants were hunted for ivory to enhance external trade (Matutu 2017). Raiding cattle and food from other tribes and collecting tributes helped sustain FS in the Monomotapa kingdom (Magama 2014). Payment and collection of tribute were an FS strategy for both the tribute payer and the collector. Despite its usage of various FS strategies, challenges emerged during the Munhumutapa era (Magama 2014; Matutu 2017). Civil strife (internal and with Sotho-Tswana people), overgrazing, a decline in external trade, successive droughts, exhaustion of the soil and pests compromised the kingdom’s FS (Magama 2014). The Monomotapa kingdom fell and paved the way for the Ndebele kingdom.

Like their predecessors, the Ndebele kingdom used various strategies to secure their FS. Chief amongst these were cattle breeding, cropping, food storage and preservation strategies (Andreucci 2018; Ndlovu Gatsheni 2009). Drought mitigation was using livestock, which the Ndebele moved from one grazing land to another in search of better grazing (Magama 2014). The king also adopted *isiphala* or *zunde* concepts to bank food (Lunga & Musarurwa 2016) and the practice of lending cattle to his subjects. These strategies assured the availability of food (Matutu 2017). Harvested crops were preserved and stored in granaries and a big pit dug in the cowshed or cattle kraal (*umncatsha*) for use during lean seasons (Iliffe 1999; Mpofu 2015). Granaries were built on huge rocks as a measure of managing and controlling moisture levels, with other crops (pumpkins, melons) being stored underneath. Local wisdom on the life cycles of pests and pest control systems were critical for sealing granaries (Chirimuuta & Mapolisa 2011). The initiatives mentioned above ensured that households could keep their products free from insect damage, ultimately protecting themselves from food insecurity.

The magnitude of most of the practices and strategies presented above was insignificant to guarantee the production of enough crops to address macro food shortages (Lunga & Musarurwa 2016; Ndlovu-Gatsheni 2009). Lunga and Musarurwa (2016) argue that the *isiphala* practice lacked active community decision-making and was void of nutrition targets. Matutu (2017) states that ordinary people supplemented food stocks through barter and trade, and the Ndebele resorted to raiding other groups for cattle and grains as a survival tactic (Ndlovu-Gatsheni 2009). Despite the two dynasties’ abilities to spread the risk as a way of ensuring their national food securities, they were not immune to challenges (see Table 2). Colonisation in 1890 collapsed the Ndebele dynasty.

### Colonial period (1890–1980)

The colonial period refers to the period of British occupation of Zimbabwe, between 1890 and 1980 (Baxter 2010), whereby indigenous Zimbabweans were dispossessed of their land. Several land policies, segregation and violence were the order of the day (Blake 1977; Mutasa 2020). According to Raftopoulos and Mlambo (eds. 2008), the colonial period ushered in capitalism, which transformed social and economic relations. Early modern mines, towns, new forms of struggles and identities emerged. Various policies consolidated the colonial rule and alienated two-thirds of the indigenous people from agro-ecological regions I–III to drought-prone regions IV and V (Maruve & Chitongo 2017), triggering livelihood insecurity and resource-based conflicts. Some of the policies bear reference to the *Land Apportionment Act*, the *Masters and Servants Ordinance*, the Rhodesia Native Labour Bureau, Pass Laws, the *Native Regulations Ordinance* and the compound system that gave mine owners semi-feudal powers akin to those of slave owners of the 19th century (GOZ 2020). The colonial period’s policies ignited the liberation philosophy that presented in various forms of resistance strategies, leading to Zimbabwe’s independence in April 1980. Zimbabwe’s attainment of independence marked the end of the colonial period.

Food initiatives in the colonial period centred around agricultural extension services and the Grain Marketing Board (GMB) mechanism (Kramer 1997). These extension services were used to bring in new information and technologies to improve farmers’ attitudes, production skills, incomes and standards of living (Azumah, Donkoh & Awuni 2018). The extension services disseminated information and developed food production skills to improve the living standards of beneficiary communities using four approaches, namely:

1. The technology transfers extension (TTE)
2. The advisory extension
3. Facilitation for empowerment
4. Master farmer training (MFT).

The TTE was predominantly used in the 1960s and 1970s by the then-government (Masere 2015) to prescribe seed varieties and practices to farmers at reduced costs (Kumar & Sharma 2018). However, because of its coercive nature, the targeted farmers rejected it, deeming it to be a punishment from colonial masters (Masere 2015). In the same period, the colonial government used the advisory extension approach to promote interaction between the government, private consulting companies and farmers to offer farmers technical advice and prescriptions (Kumar & Sharma 2018). The facilitation for empowerment extension approach was used to train lead farmers to produce higher yields. A study in the Manicaland and Masvingo provinces of Zimbabwe found that only 43% of the sampled farmers used this extension approach (Dube 2017). Later, the colonial government employed the MFT extension approach in the 1930s (Pazvakavambwa 1994) to improve smallholder agriculture using new farming techniques. Farmers who demonstrated the mastery of extension skills were used to pass on the skills and information to other farmers through demonstration (Hemmes & Vissers 1988). The philosophy of MFT was meant to promote agricultural skills development and innovation amongst farmers. The agricultural extension paved the way to the other FS strategy, the GMB. The GMB’s institution was triggered by the 1930 world recession, mainly to address food insecurity challenges through marketing agricultural products, maize and wheat (Matsive 2012). The GMB concept was a recession-induced government response to ensure national FS, by the same token with *isiphala* and *zunde* concepts of the precolonial period in terms of purpose. Zhou (2012) indicates that a total of 178 900 metric tons were imported in response to the 1946–1947 crop failure, whilst 143 500 mt were imported after the 1950 drought to ensure national FS. Matandare (2017) posits that GMB extended its buying depots in the small farm areas in 1975. The colonial period ended after 1980 when Zimbabwe gained independence.

### Postcolonial period (1980 to present)

In 1980, Zimbabwe became Africa’s newest independent state (Riddell 1984), seeking to redress colonial-period imbalances by assimilating previously marginalised people into the mainstream economy (Sibanda & Makwata 2017). The GOZ and its development partners came up with several economic blueprints and FS initiatives aimed at promoting sustainable economic growth and poverty alleviation. Raftopoulos and Mlambo (eds. 2008) present that a range of political and economic convulsions saw the emergence of new social-related and state authoritarianism and dispossession of people that will be unpacked in the following sections.

## Methodology

The study used a qualitative paradigm to gain an impression of how Zimbabwe ensured its FS from the years 1430 to 2020 and to interrogate the country’s FS initiatives. This interpretive design was adopted for its ability to systematically and objectively describe life and give meaning to human experiences (Patel & Patel 2019) and understand meanings that the people of Bulilima, Gwanda, Mangwe and Umzingwane districts attach to the FS phenomenon. Data were provided by a four-tiered qualitative data collection methodology, namely literature review, participatory observation, questionnaire and focus group discussions. A desktop study was conducted to collect data from secondary sources. It consisted of reading and extracting information from government reports, scientific journal articles, NGO reports, FS policies, UN reports and policy briefs. Secondary data review was meant to determine and ascertain the most current developments in FS and provide insight into past and current FS efforts and challenges. The information from the desktop review was used to triangulate with empirical data collected through fieldwork observation, structured questionnaires and focus group discussions. Purposive sampling was used in the selection of participants and research sites. The selected research sites were Bulilima, Gwanda, Mangwe and Umzingwane districts in Zimbabwe. The districts are similar with respect to social and cultural aspects and susceptibility to hazards, thus providing rich opportunities to observe the positive and negative aspects related to FS closely. A total of 85 participants comprised district development coordinators, the Environmental Management Agency, Rural District Council chief executive officers, councillors, traditional leaders (chiefs), NGO managers and heads of schools. The participants had decision-making, institutional gatekeepership and custodianship of communities, knowledge and technical expertise and experience in the implementation of development projects. Qualitative Research Software (QSR) NVivo was employed in data analysis to come up with themes and to identify patterns in the data (Archer 2018); it was chosen for its efficiency and ability to compare different codes.

### Ethical considerations

Ethical clearance was obtained from the North-West University (NWU) (reference number NWU-01665-20-A9).Ethical standards were followed by explaining the purpose of the research and by giving participants the assurance that confidentiality would be maintained. Participants were assured that the information they provided would be used solely for educational purposes.

## Findings and discussions

This section focuses on the presentation of the data collected in the field, zeroing in on FS initiatives in Zimbabwe.

The participants had three distinct age range categories. About 29 respondents were aged between 18 and 35 years, 32 were in the 36–50-year-old range and 24 were over 51 years old. The average age of the study participants was 31 years, and the oldest participant was 84 years old. The majority were male (55%) and 45% were female.

### Food security initiatives undertaken in Zimbabwe

Government officials interviewed explained that Zimbabwe’s FS initiatives span over three periods, the precolonial, colonial and postcolonial. Of the three periods, the postcolonial period has seen an increase in the number of players and investments working towards the implementation of FS initiatives. Some of these players are the GOZ, the FAO, the World Food Programme, World Vision International, the Organization of Rural Associations for Progress and Compassionate Development Services, amongst others. This was also corroborated by NGO staff who were involved in FS programs in the country. One NGO staff member had this to say during in-depth interviews:

‘Our organization is implementing livelihoods and food security programs using technology to increase yields and prevent serious food insecurities in moments of climate variability; CA [*conservation agriculture*] scaling up programed banking through the provision of seed; fodder production through hay bale production training, and taking social service ministry to the communities.’ (Interviewee 41, aged 54 years, is NGO program manager)

Affirming the plurality of FS initiatives during the postcolonial period, the Umzingwane district focus group discussion (FGD) meeting echoed:

‘We have DRP [*Drought relief programme*]; command livestock agriculture, now called smart agriculture; cash transfer programs; seed bank; small livestock; nutrition gardens; water and sanitation [*WASH*]; food for assets programme [*people work for something in return*]; school feeding programmes; subsidised stock feed program and free tillage program for the vulnerable.’ (FGD4, female, aged 43 years)

It was highlighted during FGDs that projects targeted the vulnerable members of the community and individuals who showed interest in the projects, especially those that build resilience, empower the communities and make them feel like being part of whatever is happening in the area to develop a sense of ownership.

Observation during data collection revealed that about 2020 scores of FS-related projects had varying impacts and had been implemented in most districts of Zimbabwe. Participants noted that many NGOs were duplicating programs in bits and pieces and brought a lot of confusion. The number of implemented projects was too high and geographically spaced to show a significant impact on communities. Observations and perceptions of the participants revealed that agriculture-related projects, feeding programs, poverty eradication and drought relief programs were the most popular. Drawing from these findings, earlier history initiatives in the precolonial states have been reinvented in new names and refreshed processes. As a result, a new multistakeholder approach to FS has been born, whereby the government has built partnership with various implementing partners (NGOs). This new approach has seen the government implement massive agriculture-related projects, whilst the NGOs focused on capacity-building and food distribution, respectively. In general, respondents understood FS initiatives existed to build community resilience, ensure FS, socio-economically empower communities (especially women and the vulnerable), eradicate poverty, capacitate the community, safeguard national assets and develop a sense of program ownership. Table 1 is a summary of the findings derived from the interviews.

**TABLE 1:** Implemented projects and aims or objectives.

Focus group discussion		Implementing partners (NGOs)	
Projects	Aims or objectives	Projects	Aims or objectives
Agricultural projects	Build resilience	Agricultural programmes	Capacity building
Command agroforestry	Develop a sense of ownership	Basic entrepreneurship	Women’s empowerment
Developmental fund	Empower community	Capacity building	Improve technologies and practice
Drought relief programmes	Socio-economic empowerment	Drought relief	Irrigation for access to food
Feeding programmes	Ensure FS	Financial literacy	Mentoring
Food for assets		Fodder crop project	Poverty eradication
Poverty eradication		Food distribution	Reaction to El Niño
Seed bank		Small grain project	Build resilience
Water and sanitation		Social service ministry	Minimise CF and livestock conflicts
		Technical skills	Ensure FS

NGOs, non-governmental organisations; FS, food security; CF, crop failure.

Mabhena (2013) adds a new dimension that the postcolonial FS initiatives addressed poverty, FS and people’s living standards, to which the earlier historical period paid little attention. Therefore, Mabhena’s assertion is closer to the FAO et al. (2017) definition of FS, which is physical, social and economic access to sufficient, safe and nutritious food to meet the community’s dietary needs and food preferences. Deducting from Table 1, agricultural production was the mainstay of FS across all the three historical periods and is viewed to be key in achieving FS at any level. Meanwhile, the storage and preservation strategies (using granaries, *zunde* or *isiphala* and the GMB) that were also used cut across the three historical periods to mitigate food shortages in the event of calamities. Conversely, capacity development and socio-economic empowerment were viewed as key facets for ensuring long-lasting FS during the colonial and postcolonial periods. Drawing from the above discussions, the perception of FS has evolved from being mainly a household worry to a government and international concern. As a result, all forms of commitment in terms of policies, budgetary, support and resources have been mostly observed during the postcolonial era (Echanove 2017; Mutukura 2015; Nkala 2016).

Furthermore, desktop study findings, as well as those of participants (especially from the government side) and traditional leaders, revealed that some of the postcolonial-period FS initiatives (postcolonial, i.e. from 1980 to present) are the following:

- Drought relief programmes (DRPs)
- Land Reform and Resettlement Programme (LRRP)
- Command Agriculture
- Zimbabwe Agenda for Sustainable Socio-Economic Transformation (ZimASSET) (Government of Zimbabwe (GOZ) 2013b)
- Zimbabwe’s food security and nutrition policy (FSNSP).

#### Drought relief programmes

According to Munro (2006), the Drought relief Programme (DRP) strategy was perceived as an effective rational, organised and controlled response to a food crisis, hence its usage to reduce the impact of droughts in Zimbabwe. Maxwell, Russo and Alinovi (2012) adds that the main purpose of DRPs is to protect and promote the livelihoods of poor and vulnerable households in the context of a prolonged crisis. Some components of the DRPs’ strategy bear reference to conservation agriculture, cash for assets, village savings and lending, nutrition gardens and small livestock pass-on schemes (Jennings et al. 2013). Similarly, Sazali (2015) postulates that studies conducted between 2003 and 2015 in Zimbabwe show that livelihood interventions improved livelihoods, built resilience and stimulated rural development. However, Jennings et al. (2013) contend that the extent to which the interventions contributed to the impact was unclear, as the indicator may have picked up on the benefits of economic recovery and/or the effects of inflation. Therefore, the DRPs serve as a drought impact reduction strategy that works towards saving the livelihoods of the poor and vulnerable households, at the same time building their resilience and stimulating rural development. Later, LRRPs were initiated.

#### The land reform and resettlement programme

Mutasa (2020) stresses that Zimbabwe’s LRRP in 2000 sought to correct the colonial period land ownership imbalances. The LRRP changed land ownership laws, regulations and broadened agricultural production (Maruve & Chitongo 2017). The LRRP was twofold, that is, LRRP I from 1980 to 1998 and LRRP II, the Fast-Track Land Reform Program (FTLRP), since 2000 (GOZ & World Bank 2019). Land Reform and Resettlement Programme I was based on the willing buyer–willing seller approach, whereby the government bought white people’s commercial farms for redistribution, whilst FTLRP was a state-led accelerated approach for appropriating land from white farmers without a resort to courts for compensation for both A1 and A2 resettlement models (Mutasa 2020). Echanove (2017) asserts that the 2000 land reform had mixed results. On the positive side, the GOZ has resettled 350 000 indigenous families under the A1 and A2 models on 14.4 million hectares since independence (Maruve & Chitongo 2017). The LRRP helped decongest the marginal communal areas (Mutasa 2020). Despite the FTLRP’s pivotal role in decongesting communal lands and alteration of land ownership dynamics, it led to a 20% decline in agricultural production (Mutasa 2020). The new farmers did not have the capital to maintain the previous levels of production white commercial farmers used to reach (Mutasa 2020), hence its negative impact on FS. Therefore, land ownership without adequate capital to maintain agricultural production is not the panacea to productivity but a recipe for food insecurity. The LRRP preceded the command agriculture scheme supported by Sakunda Holdings and spearheaded by the army.

#### Command agriculture

Command agriculture refers to a contract farming scheme rolled out in 2005 after the land redistribution, as well as a strategy to combat food shortages (Makuwerere Dube 2020). Moyo and Nyoni (2013) clarify that this initiative was guided by the thinking that improved logistics would result in timely delivery of inputs, hence the attainment of self-sufficiency in terms of FS through production and strengthening of the national strategic grain reserves (Nkala 2016). Selected A1 and A2 farmers were contracted to produce a set amount of the staple maize crop to ensure food self-sufficiency (Mutonhori 2017). Mutonhori (2017) opines that this strategy can be beneficial but criticises the use of the term ‘command agriculture’ as a bit clumsy, because it depicts military and command economies. Years later, in 2013, a new FS initiative was established towards strengthening economic sustainability with more FS policy and sustainable transformation initiatives.

#### Zimbabwe’s food security and nutrition policy

Echanove (2017) asserts that Zimbabwe’s institution of the FSNP in 2012 indicates commitment and obligations to end hunger and reduce malnutrition. This policy outlines FS stakeholder roles, responsibilities and principles (Government of Zimbabwe 2013a). Principles reaffirm the right to food; ensure contextual socio-economic relevance, evidence-based best practices, strengthening and reinforcement of sectoral collaboration, partnerships, roles and responsibilities; reaffirm that relief, recovery and development occur simultaneously; and foster multisectoral approach in assessment, analysis and action. Zimbabwe’s FSNP commits to policy advice and analysis, agriculture and FS, social assistance and social protection, food safety and standards, nutrition security and food and nutrition security information to ensure national FS. The FNSP mandates lead agencies and ministries to guide and facilitate the policy implementation in explicit strategic objectives, actions, outputs and outcomes (Food and Nutrition Council [FNC] 2014). The FSNP is evaluated through a three-phase monitoring framework (Mukudoka 2013). The first phase focuses on activity and output that monitors the levels of FS plans for province and district capacity and the performance of the Food Nutrition Security Council quarterly. Meanwhile, the second level monitors the outcome on the level of commitments of the lead ministry annually. The establishment of an FSNP and a monitoring and evaluation (M&E) framework emboldened Zimbabwe’s commitment to FS. A year after the inauguration of the FNSP, Zimbabwe crafted a policy towards transforming and strengthening economic sustainability.

#### Zimbabwe agenda for sustainable socio-economic transformation (2013–2018)

The ZimASSET is a broad-based socialist policy towards economic sustainability, transformation and strengthening through the full exploitation of internal relationships and linkages of various facets of the economy (Sibanda & Makwata 2017). The facets of the economy bear reference to FS and nutrition, social services and poverty eradication, infrastructure, utilities, value addition and beneficiation. The main object of the initiative was economic revival and wealth creation, anchored on indigenisation, empowerment and employment creation, as well as exploitation of the country’s abundant human and natural resources (Mangwana 2014). These cluster programmes are aligned to and informed by the Comprehensive African Agricultural Development Programme, the Draft Comprehensive Agriculture Policy Framework (2012–2032), the Food and Nutrition Security Policy, the Zimbabwe Agriculture Investment Plan (2013–2017), SADC and the Common Market for Eastern and Southern Africa (COMESA) Food and Nutrition Frameworks (GOZ 2013b). The ZimASSET concludes the post-independence FS initiatives. The section below draws attention to challenges that have been experienced during the historical phases of Zimbabwe’s FS efforts and initiatives.

### Challenges for Zimbabwe’s food security initiatives

The study found that the three historical periods were faced with impediments that each posed their own unique challenges in their given timeframes. See a visual historical footprint of these phases in Table 2.

**TABLE 2:** A historical visual footprint of challenges for Zimbabwe’s food security initiatives.

Challenges	Period		
	Precolonial 1430–1885	Colonial 1891–1980	Postcolonial 1980–2020/1
Bureaucracy	-	❖	-
Civil strife	❖	❖	-
Corruption	-	-	❖
Decline in external trade	❖	-	❖
Exhaustion of soil	❖	-	-
Incapacitation	-	❖	❖
Non-repayment of loans	-	-	❖
Pest and animal diseases	❖	❖	❖
Political antagonism	❖	❖	❖
Poor planning and supervision	-	-	❖
Scepticism	-	❖-	❖
Successive droughts	❖	❖	❖

*Source:* Kramer (1997), Nkala (2016), Andreucci (2018), Gavin (2021).

Note: Please see the full reference list of the article, Ngwenya, S., Lunga, W. & Van Eeden, E.S., 2022, ‘Learning from past and current food security efforts and challenges in Zimbabwe: The years 1430–2020’, *Jàmbá: Journal of Disaster Risk Studies* 14(1), a1210. https://doi.org/10.4102/jamba.v14i1.1210, for more information.

Drawing from Table 2, civil strife dominated the precolonial and colonial periods whilst soil exhaustion was equally prevalent at the time. Using fertilisers was a dream far in the future at that time. Kramer (1997) affirms that bureaucratic tendencies impeded the colonial period’s FS initiatives. The study found that the outbreak of pests, livestock diseases and political antagonism have continued to destabilise FS initiatives in Zimbabwe from as early as the precolonial period to the postcolonial period Zimbabwe. Therefore, these challenges have led to economic decline and great food insecurities, hence the need for a lasting solution for these phenomena. However, Mutasa (2020) opines that political antagonism between Zimbabwe and the West during the postcolonial period imploded the country’s FS initiatives, crippling its economy and making it difficult for farmers to access inputs. In the same manner, Mangwana (2014) advances that postcolonial FS initiatives like ZimAsset and command agriculture were met with scepticism and downright negativity because they were perceived to be partisan, noninclusive and over-ambitious. Consequently, they suffered a lack of buy-in by critical stakeholders (Nyoni 2017). In the same view, non-repayment of loans by project beneficiaries led to the demise and abandonment of the postcolonial period FS initiatives (Nkala 2016). Poor planning, supervision and service delivery, weak policy implementation discipline (Nyoni 2017) and ineffective monitoring systems (Irigoyen 2017) affected the implementation of the FS initiatives in the same period. More so, financially related corruption practices jeopardised FS initiatives in the postcolonial period (Matandare 2017). Furthermore, this finding is affirmed by 2019 Transparency International Corruption perceptions index that ranks Zimbabwe as the 21st most corrupt country in the world (Transparency International 2020). Corruption malpractices point to unscrupulous politicians that stole and diverted program inputs from projects (Gavin 2021; Sibanda & Makwata 2017). Drawing from the visual historical footprint summarised in Table 2, FS will remain far away from being a reality in the lived realities of Zimbabwe, unless the identified challenges (such as poor planning, supervision and service delivery, weak policy implementation discipline, ineffective monitoring systems and corruption) are tackled head-on by all stakeholders (Nyoni 2017; Samukange 2015).

## Concluding remarks

It is apparent from the discussions that various international and national strategies have been employed to ensure and secure FS for all people in Zimbabwe. Studying Zimbabwe’s three historical periods (precolonial, colonial and postcolonial) provides a fertile ground (lessons and practices) on which new FS programs can be designed and modelled. The study further noted an increase in the number of FS players and strategies during the colonial and postcolonial periods; this could be influenced by the returns that come with implementing FS programs and projects. Consequently, this increase resulted in duplication of programs and antagonism amongst some stakeholders between 1980 and 2020, hence the demise of the FS initiatives during these periods especially. The study also found that despite a decorated history of FS strategies, Zimbabwe’s FS initiatives across the three historical periods between 1430 and 2020 remain susceptible to various challenges. These challenges can be categorised as family (strife, soil exhaustion, diseases), power (bureaucratic tendencies, lack of project buy-in by critical stakeholders, poor management, weak policy implementation discipline) and in recent times, the 2020 corruption malpractices, amongst others (Kairiza & Chingono 2019; Matandare 2017). Thus, the country’s food insecurity by 2020 remained far away from being a lived reality unless the identified challenges were tackled head-on by all stakeholders. Another observation was that the limited impact of FS initiatives, punctuated with food insecurity recurrences, pointed to stakeholder exclusion and nonparticipation in all stages of the project cycle. Informed by local wisdom developed across the three historical periods, Zimbabwe’s food insecurity challenges could be overcome by adapting, organising, cultivating the FS culture and strategies like the *isiphala* or *zunde* concepts to bank food, food storage, moisture management and controls and pest control systems, amongst others. Practising and sharing these strategies with the younger generations will help preserve this local wisdom and ensure FS over a long period.

Based on these findings and conclusions, the study recommends the GOZ and its development partners pursue and enforce the reduction of the FS implementing players, so as to bring sanity, order and objectivity in the FS arena. An investigation was to be made on the sudden rise of interest in FS by many players. For FS to be a lived reality, all proposed changes should be implemented forthwith, with informed local wisdom being given space in finding a lasting solution to food insecurity by all FS stakeholders (GOZ, donors and implementing partners). As such, it should be adopted by all FS stakeholders and communities that already use it, be revived and be promoted to mitigate current and future food insecurity; that stakeholder participation, knowledge development and management should be made a top priority in all FS-related efforts. Based on the scholarly works of Marakas (1999) and Bhatt (2000), in which ‘knowledge creation ensures an organisation’s sustainability and survival’, the study recommends that all development practitioners should make knowledge development and management the crux of all FS-related programs and projects to inform future programming. These will promote storage of all assessment data that work towards preserving history, best practices, lessons learned and knowledge memory of development initiatives and strategically lobbying for all stakeholder commitment through the mobilisation of political systems and institutions to commit all forms of resources to capacitate farming communities. This could foster new partnerships and encourage the ethic of working together and participation towards ensuring FS. Therefore, taking this route may be critical in addressing commitment-related food insecurity challenges. It is hoped that this research will help facilitate FS knowledge development and management to provide long-lasting FS solutions.
